# The Link between Musculoskeletal Pain, Lifestyle Behaviors, Exercise Self-Efficacy, and Quality of Life in Overweight and Obese Individuals

**DOI:** 10.4172/2329-9096.1000255

**Published:** 2015-01-20

**Authors:** Pouran D Faghri, Winnie SY Chin, Tania B Huedo-Medina

**Affiliations:** 1 University of Connecticut (Storrs), Department of Allied Health Sciences, USA; 2 University of Connecticut (Storrs), Department of Statistics, Centers for Disease Control and Prevention (CDC), University of Connecticut Center for Environmental Heath and Health Promotion, USA

**Keywords:** Overweight, Obesity, Musculoskeletal pain, Physical function, Quality of life, Exercise self-efficacy

## Abstract

**Objective:**

To determine the extent musculoskeletal (MS) pain in the low back and knee (weight-bearing (WB) joints), shoulder and wrist (non-weight bearing joints), and exercise self-efficacy mediates associations between overweight and obesity levels based on BMI (4 levels: overweight, obese class I, II, or III), physical function, emotional role, social interference, and physical activity (PA) levels.

**Design:**

Cross-sectional study.

**Setting:**

Four long-term nursing home facilities in the Northeast U.S.

**Participants:**

99 overweight or obese (BMI > 25) nursing home employees.

**Interventions:**

Self-reported survey administered to employees who met inclusion and exclusion criteria.

**Main Outcome Measure(s):**

General health status, physical function, emotional role, Exercise Self-Efficacy Scale (ESE), physical activity (PA), and frequency of pain at each joint.

**Results:**

Reported pain frequency were 66.3%, 54.4%, 42.2%, and 24.1% for lower back, knee, shoulder, and wrist, respectively. Higher obesity levels were associated with lower physical function (r=−0.109, p=0.284). PA decreased with higher obesity levels (r=−0.248, p<0.05), particularly in moderate PA (r=−0.293, p<0.05). Obesity was associated with a lower ESE (r=−0.239, p<0.05). Wrist pain significantly mediated the effect of obesity on moderate physical function, emotional role, and ESE. ESE was a significant mediator between obesity and moderate and vigorous PA.

**Conclusions:**

Overweight and obese nursing home employees are at higher risk for developing musculoskeletal disorders due to high demand, low control jobs, and the associated biomedical compromises while working. To increase the effectiveness of weight loss interventions for this population, the mediating effects of MS pain with higher levels of obesity should be considered.

## Introduction

Within the last 20 years, prevalence rates of obesity have dramatically risen from 10% to more than 30% in the United States [1]. In the year 2015, it has been estimated that about 75% of adults will be overweight or obese, and 41% of U.S. adults will be obese [2]. Current trends show that obesity is associated with increased prevalence of adverse health conditions, including coronary heart disease, cancers (endometrial, breast, and colon), hypertension, stroke, dyslipidemia, type 2 diabetes, respiratory problems, and musculoskeletal conditions [1,3]. These conditions are also referred to as Obesity-related Comorbidities (ORCs), and have a substantial impact on morbidity rates [4]. In the U.S., approximately 280,000 to 325,000 deaths each year are attributable to obesity [5,6]. Obesity and its comorbidities are also associated with changes in health-related quality of life (HRQOL) in overweight and obese individuals, an outcome involving substantial decreases in physical and mental health [3,7–15].

Increased adiposity in overweight and obese individuals are associated with mechanical and structural changes that may impact physical health and induce musculoskeletal conditions [3,8,16–19]. Higher Body Mass Index (BMI) may lead to increased loading on individual joints and localized inflammation, which contribute to muscle and bone loss, joint misalignment, and postural changes [3,17,19–23]. These biomechanical and physiological alterations add to the development of pain, musculoskeletal conditions and joint disease [3,8,24]. Musculoskeletal pain in various body sites have shown positive associations with increased BMI [25–35]. Understanding the mediating effect in Weight-Bearing (WB) versus Non-Weight-Bearing (NWB) joints is needed to expound the role of pain in the BMI-HRQOL relationship.

Previous research has shown strong associations between obesity and self-efficacy, defined as an individual’s confidence in their ability to perform a specific behavior to achieve a particular outcome [36]. Individuals with a high BMI consistently report a lower exercise self-efficacy (ESE), suggesting less confidence in their ability to change or keep up with an exercise regimen. Decreases in ESE are positively correlated with decreases in physical activity (PA) levels [32,37]. In recent weight-loss interventions that targeted ESE, modest to large weight reductions of 5–10 kg led to significant increases in ESE [38,39]. However, these findings need to be further elucidated through statistical models that posit ESE as a mechanism by which obesity affects PA level [32,35,39,40].

ORCs have been tested as potential mediators that explain part of the adverse effect of obesity on HRQOL outcomes [7,13,15,32–36]. J-shaped associations between obesity and BMI indicate that lower HRQOL was observed in those with abnormal body weight (underweight, overweight, and obese individuals) [5]. In particular, pain in 2–4 sites partially mediated about 22–44% of the association between obesity and HRQOL, suggesting that obesity’s adverse effect on HRQOL may be manifested through musculoskeletal pain [13]. Explanatory models that estimate the musculoskeletal pain as a mediator in this relationship would be useful in the development of future interventions for the overweight and obese population [24,41].

The present study seeks to evaluate the prevalence of musculoskeletal pain in a group of overweight and obese individuals and to identify the mediating effect of pain and ESE in the relationship between obesity, PA, and specific HRQOL items. We hypothesize that: 1) musculoskeletal pain prevalence will be high in both WB and NWB joints; 2) increased obesity will indicate lower HRQOL; 3) pain will partially mediate relationships between BMI, HRQOL, and PA; and 4) ESE will partially mediate the BMI-PA relationship.

## Materials and Methods

### Design

A cross-sectional observational study.

### Participants

The study sample included ninety-nine overweight and obese nursing home employees who were at risk for type 2 diabetes based on diabetes risk score >8, indicating that risk is high for having pre-diabetes presently [42,60]. Participants were recruited from four long-term care facilities located in the Northeast United States, and were required to sign an IRB-approved consent form.

### Measures

#### Body Mass Index (BMI)

Trained health educators measured height (nearest mm) and weight (nearest 0.1 kg) using a calibrated Seca 700 physician balance beam scale. BMI was calculated as weight (kg) divided by height (m^2^) and categorized based on CDC recommendations of overweight (25–29.99 kg/m^2^), obese class I (30–34.99 kg/m^2^), obese class II (35–39.99 kg/m^2^), and (>40 kg/m^2^) [61].

#### Participant questionnaire

A standardized questionnaire was distributed to all participants. The questionnaire obtained information on demographics, Musculoskeletal Pain, Physical Activity (PA), Health-Related Quality of Life (HRQOL), and Exercise Self-Efficacy (ESE).

Musculoskeletal pain was defined as frequency of pain in the WB joints (low back and knee) and NWB joints (shoulder and wrist-forearm) and asked how often on hourly, daily, weekly, or monthly basis the participants felt pain.

Physical Activity (PA) was first assessed using a question on self-reported typical current PA not specific to work. Three other questions obtained information on performance of mild, moderate, or vigorous PA for a 30-minute duration during a typical 7-day week.

Health-Related Quality of Life (HRQOL) was evaluated with 5 survey items from the Short Form Survey (SF-12), a validated health survey. General Health Status was defined as self-reported general health using the first SF-12 question that asked how the individual perceived their health. Physical Function was assessed using the second and third SF-12 questions, and asked if the individual perceived their health to limit them in moderate activities (moving a table, pushing a vacuum cleaner, bowling, or playing golf) and vigorous activities (climbing several flights of stairs). Emotional Role was assessed using the sixth and seventh SF-12 question and asked how often the participant accomplished less than they would like and done work less carefully in their work or regular daily activities as a result of any emotional problems. Social Interference was assessed using the last SF-12 questions and asked if the individual perceived their joint problem to interfere with social activities with family or friends.

Exercise Self-Efficacy (ESE) was calculated with a summary score comprised of 11 questions from the Sallis (1998) ESE Scale. Participants were given questions regarding exercise-related activities and asked how confident they were that they could keep it up for 6 months.

## Statistical Analysis

### Expectation-maximization imputation

Data was analyzed using the SPSS software version 21.0 and utilized Preacher & Hayes (2008) Indirect Bootstrapping Macro. Composite scores were created for the ESE scale. Little’s Missing Completely at Random (MCAR) test was executed for missing data (>5%), and Expectation-Maximization was subsequently performed using SPSS Missing Value Analysis imputation to create maximum likelihood estimates for randomly missing data (p=0.265).

### Descriptive statistics and associations

Descriptive and frequency analysis were used to analyze MS pain prevalence. Variables were reverse coded if needed. Normality was assessed using histograms and frequencies for all variables and outcomes. Total exercise self-efficacy score was calculated from a series of questions. Correlational analyses using Pearson’s Correlation were used to analyze direct relationships between levels of obesity, general health, physical function, emotional role, social interference, physical activity levels, and exercise self-efficacy. Benferroni correction was calculated to restrict significance to a more conservative p-value (p=0.005) to reduce Type I error.

#### Observed (Manifest) variables and path analysis (SEM)

**Figure 1:**
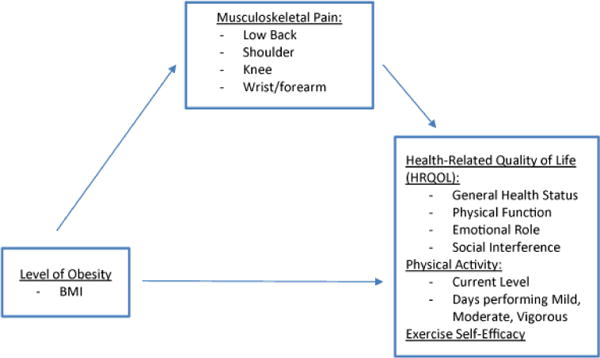
Theoretical Model of Obesity, Musculoskeletal Pain, and Health-Related Quality of Life Outcomes (OMH).

**Figure 2:**
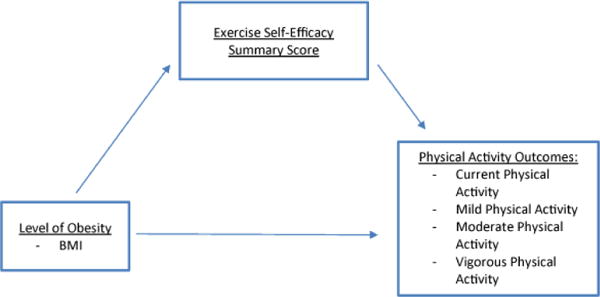
Theoretical Model of Obesity, Exercise Self-Efficacy, and Physical Activity Outcomes (OEP).

Path analysis via structural equation modeling (SEM) and bootstrapping is a useful statistical approach to examine mediation between observed variables, and was used to examine direct and indirect effects within the two theoretically driven models on this dataset. Path was first tested with the Obesity, Musculoskeletal Pain, Health-Related Quality of Life (OMH) with musculoskeletal pain in four sites as mediator variables (Figure 1). SEM was also performed for the Obesity, Exercise Self-Efficacy, Physical activity (OEP) model with ESE as the mediator variable (Figure 2). Bootstrapping at a 95% confidence interval was used to determine significance of indirect effects. Proportions of mediation were calculated using Microsoft Excel via algorithm supported by Kenny (2014).

As indicated in Figure 1, the Obesity, Musculoskeletal Pain, Health-Related Quality of life (OMH) model identifies level of obesity, musculoskeletal pain, and health-related quality of life, physical activity levels, and exercise self-efficacy. It is hypothesized that the OMH model predicts a higher level of obesity (indicated using BMI) will result in a lower self-reported HRQOL, PA level, and ESE, and that higher frequency of musculoskeletal pain will indirectly affect the relationship, resulting in a lower score of these three outcomes.

As indicated in Figure 2, the Obesity, Exercise Self-Efficacy, Physical Activity (OEP) model identifies the level of obesity, exercise self-efficacy, and physical activity outcomes. It is hypothesized that the OEP model predicts a higher level of obesity (indicated using BMI) will result in a lower self-reported PA outcome, and that a lower ESE summary score will indirectly affect the relationship, resulting in a lower self-reported PA outcome.

## Results

### Participant characteristics

Table 1 depicts participant demographics and body weight distribution.

The sample reported an overall pain prevalence of 68.7% in the low back, 55.6% in the knee, 47.5% in the shoulder, and 29.3% in the wrist/ forearm region.

**Figure 3:**
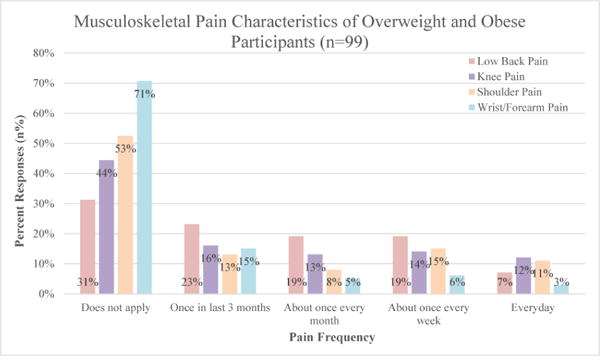
Musculoskeletal Pain Distribution among Participants.

Figure 3 indicates the frequency of WB and NWB joint pain among participants. Joint pain resided dominantly in the WB joints for weekly and monthly occurrences. However, when considering daily frequency, participants reported that the dominant joint pain sites resided in the knee and shoulder (Figure 3).

### Associations between Obesity, HRQOL, PA, and ESE

Table 2 shows Pearson’s correlations between the level of obesity and HRQOL, Physical Activity Levels, and Exercise Self-Efficacy.

There was significance negative relationship between obesity and physical function for vigorous activities (r=−0.308, p<0.05) suggesting that as obesity increased, physical function in climbing stairs, decreased (Table 2). Though significance was not achieved, a negative relationship with moderate physical function was also shown (r=−0.109). Additionally, associations between BMI and current physical activity, moderate physical activity, and exercise self-efficacy were also significant (p<0.05) (Table 2). Obesity had a significant negative relationship with current PA (r=−0.248, p=0.013), and moderate PA (r=−0.293, p=0.003). Additionally, the relationship between obesity and mild PA (r=−0.129, p=0.202) and vigorous PA (r=−0.176, p=0.082) were also negative, but did not reach significance.

### Path analysis and structural equation model

#### Obesity-musculoskeletal pain-health related quality of life (OMH) Model

Out of the 11 models used to assess the multiple variables under the theoretical OMH model, the four models that tested wrist pain as the mediator between level of obesity, physical function, emotional function, and exercise self-efficacy, indicated significant indirect effects and are presented in Tables 3–7.

Table 3 depicts the mediation results between level of obesity (indicated using BMI), musculoskeletal pain frequency in four anatomical joints, and physical function.

As shown in Table 3 the wrist-forearm joint significantly mediated the relationship between obesity and moderate physical function at about 77.7%.

Table 4 depicts the mediation results between level of obesity (indicated using BMI), musculoskeletal pain frequency in four anatomical joints, and the role of emotions in accomplishing less at work or at home.

As shown in Table 4, the wrist-forearm joint significantly mediated the relationship between obesity and emotional role in regards to accomplishing less. The proportion of mediation was approximately 35.2%.

Table 5 depicts the mediation results between level of obesity (indicated using BMI), musculoskeletal pain frequency in four anatomical joints, and physical function in a moderate activity.

Table 5 depicts the mediation results between level of obesity (indicated using BMI), musculoskeletal pain frequency in four anatomical joints, and physical function in a moderate activity.

As shown in Table 5, the wrist-forearm joint also significantly mediated the association between obesity and emotional role regarding being less careful.

Table 6 depicts the mediation results between level of obesity (indicated using BMI), musculoskeletal pain frequency in four anatomical joints, and the total exercise self-efficacy score.

As shown in Table 6, the wrist-forearm region significantly mediated the association between obesity and ESE of about 17.7%.

#### Obesity-Exercise Self Efficacy-Physical Activity (OEP) Model

Out of the four models used to assess the multiple variables under the theoretical OMH model, the three models that tested exercise self-efficacy as the mediator between level of obesity, current physical activity, moderate physical activity, and vigorous physical activity indicated significant indirect effects and are presented in Table 7.

Table 7 depicts the mediation results between level of obesity (indicated using BMI), the total exercise self-efficacy score, and levels of physical activity.

ESE had significant indirect effect on the outcomes of current PA, moderate PA, and vigorous PA. For current physical activity, a 16.44% mediating effect was indicated, while a 26.42% proportion of mediation occurred in moderate PA. Lastly, ESE had the largest proportion of mediation on vigorous PA at about 44.8% (Table 7).

## Discussion

This study aimed to understand associations between obesity and specific health-related quality of life items, physical activity levels, and exercise self-efficacy, in addition to testing two theoretically driven statistical models; the OMH and OEP models.

Our results are in line with previous research and support our first hypothesis of a higher prevalence of WB and NWB musculoskeletal pain in overweight and obese nursing home employees compared to general nursing home employee population. Comparatively, our samples’ prevalence rates are higher compared to general nursing home employees. In a study by Miranda et al., their cohort of 344 nursing home workers indicated the prevalence of musculoskeletal pain to be 34% in the low back and approximately 25% each in the knee, wrist/hands and shoulder region [56]. Another survey on nursing home personnel indicated a pain prevalence of 50.5% in the low back, 25.8% in the knee, 27.9% in the shoulder, and 17.8% in the wrist [62]. Higher prevalence of pain in all joints may be explained by associations with increased BMI [62–66].

Associations between obesity and HRQOL outcomes directionally supported the second hypothesis, with significance found in the negative relationship between obesity and physical function for vigorous activities, suggesting that as obesity increased, physical function in climbing stairs, decreased. Though significance was not achieved, a negative relationship with moderate physical function was also shown (Table 2). These results are in line with previous research reporting stronger associations with higher BMI and physical components of the SF-12 health profile [5,14,15,62–67]. In particular, Yamakawa and colleagues, showed that ambulation, a functional activity, is negatively related to obesity, supporting our results. In addition, negative relationships were expressed between obesity and general health (r=−0.191). The findings from Heo et al. support our general health association, reporting that compared with desirable weight adults, underweight, overweight, and obese adults were significantly more likely to report poor to fair general health status.

An increase in emotional role limitations and social interference (r=0.137 and r= 0.004, respectively) were shown coincident with higher BMI, although associations were not significant. These results are supported by the results of Jia & Lubetkin that show HRQOL scores are significantly lower for overweight and obese participants. Several other studies support these results that there is a direct relationship between level of obesity (indicated using increased BMI), and greater HRQOL impairment [5,14,15,33,67,68]. Similar associations are reported in other studies between obesity and depression and/or mental health impairment [10,69–71]. Specifically, Jia & Lubetkin showed that mental component scores on the SF-12 were most impaired at the extremes of BMI (underweight and obese class III), further supporting the positive association within our data. Additionally, the National Obesity Observatory (NOO) has indicated that several studies posit a bi-directional relationship between mental health and obesity, in that lower mental health may contribute to obesity, and higher obesity may contribute to lower mental health [72].

Obesity had a significant negative relationship with current PA and moderate PA (Table 2). Additionally, the relationship between obesity and mild PA and vigorous PA were also negative, but did not reach significance (Table 2). These results are supported and explained, in part, to the mechanical-structural changes that have been proposed to cause decrease in ambulation and change in gait patterns [3,25,29,45]. Hills and colleagues reported that obese individuals had changes in foot structure, with higher plantar pressure under their longitudinal arch and on metatarsal heads during both standing and walking, making it more likely to feel discomfort [73]. Previous literature have also reported reductions in knee range of motion [18,31,74]. Overweight and obese individuals have been shown to walk with shorter step length, lower cadence, and lower velocity due to excessive adipose tissue on the inside of the thigh [31,74]. Yamakawa et al. and a systematic review by Nantel support that walking is indicative of increasing PA level and mobility. Therefore with increased weight, ambulation becomes more energy intensive and uncomfortable, resulting in further sedentary behavior and obesity [25]. Though ambulation was not directly assessed in our study, PA decline may be attributed to similar mechanisms. In addition, aerobic capacity in obese individuals has been shown to be lower than normal weight individuals [75]. These mechanisms proposed by previous researchers offer some support for our results that increases in obesity may result in a decrease of PA level.

ESE expressed a significant negative relationship with obesity (Table 2). This trend is supported by current as literature in that an individual’s BMI increased, their level of ESE decreased [32]. While we did not assess postmenopausal prevalence, the majority of the participant population was 30–49 years old. This suggests a need for addressing exercise self-efficacy in this population in order to increase an individual’s confidence that they can start or keep up exercise and lose weight, to counter the effects of being overweight or obese. Trost and colleagues found significant results indicating that their obese participants were significantly less confident in their ability to overcome barriers to physical activity, to ask parents to provide opportunities for physical activity, and to choose physically active pursuits over sedentary ones [32].

In our third hypothesis, the mediated structural hypothesis of the OMH model was supported for partial mediation in some outcome variables. Wrist-forearm pain, significantly mediated (95% CI: −0.1050, −0.0036) about 29.7% of the relationship between obesity and moderate physical function (Table 3). This suggests that the adverse effects of obesity on moderate physical function may be manifested in part, through wrist-forearm pain. Since our population was primarily female (approximately 91%), these results closely align with findings from Fowler-Brown and colleagues, which showed a 22–44% partial mediation of bodily pain with physical function in women. Furthermore, their study found that bodily pain was significantly associated with disability, performance, and physical function [13]. A study by Heo et al. found that attenuation in the association between obesity and functional impairment was largely explained by medical comorbid conditions, and indicated the presence of joint pain and mental health problems further reduced the odds ratios in this relationship [10].

The wrist-forearm joint also significantly mediated 35.2% of the relationship between the role of emotions in accomplishing less at work and at home (95% CI: 0.0010, 0.1520; 95% CI: 0.0015, 0.0985) (Table 4). Pain has been supported to be a deterrent to activities of daily living (ADLs), and has been supported by recent reviews [3,8,9]. Additionally, Heo et al. found in their mediation analysis that the effects of high BMI on HRQOL were significantly attenuated when musculoskeletal pain and obesity-related comorbidities were included in the models. Other studies assessing international differences in chronic widespread musculoskeletal pain showed that excess prevalence in countries of Eastern Europe were associated with poorer physical health and psychosocial factors (stressful life events) [9,76,77]. These findings offer some support for our results that wrist-forearm pain is a critical component in the relationship between obesity and emotional role in accomplishing less at work or at home.

In assessing emotional role of being less careful at work or at home, the wrist-forearm joint significantly expressed inconsistent mediation (95% CI: 0.0015, 0.0985), with the indirect effect creating a larger direct effect than the total effect (Table 5). This suggests that the wrist-forearm may actually decrease the role of emotions in being less careful due to pain, and make the individual more careful. One potential explanation could be the fear-avoidance model [41,78], which postulates that an acute episode of pain may cause the individual to develop pain-related fear that results in attention to pain and guarded movements, however should be conditional only to those who reported wrist-forearm pain. In our sample, an acute episode of wrist-forearm pain may have resulted in more attention to activities requiring heavy use of the joint, and contribute to careful behavior. This may have long-term implications in that these individuals may develop a chronic condition due to the adaptations initiated from feeling acute pain.

Lastly, the pain in the wrist-forearm region also significantly mediated the relationship between obesity and ESE (95% CI: −0.1510, −0.0137), at about 17.6% (Table 6). This suggests the adverse effects of obesity on lower ESE may be manifested in part, due to wrist-forearm pain. Coinciding with previous research, lower ESE has consistently been associated with obesity, and is also associated with musculoskeletal pain [40]. In particular, obese females have been reported to have lower self-efficacy regarding physical activities and this must be addressed in order to increase compliance with exercise [18].

In our last hypothesis, the mediated structural hypothesis of the OEP model was supported for partial mediation in almost all variables except for mild PA. ESE significantly mediated the relationship between obesity and current PA levels (95% CI: −0.1348, −0.0001) of about 16.4%. This furthers previous research regarding obese adolescents [34,35,39] in that not only does ESE at baseline predict subsequent levels of PA, but is an explanatory variable in the relationship between increased BMI and decreased physical activity behavior.

ESE also significantly mediated the relationship between obesity and moderate physical activity levels (95% CI: −0.1595, −0.0156) with about 26.4%, as well as vigorous physical activity levels (95% CI: −0.1365, −0.0171) of about 44.8%. These results coincide and further research by Trost, Kerr, Ward, and Pate, in which obese children not only exhibited significantly lower daily accumulations of moderate and vigorous physical activity, but also significantly lower levels of physical activity self-efficacy. However, our results show that ESE is a significant partial mediator of the adverse effect of obesity on physical activity behaviors in this sample of overweight and obesity. Building confidence of overweight and obese individuals, who intend to start physical activities, even if the intensity is low, has been shown to be important specifically for females, who report lower confidence in physical activity [18].

## Study Limitations

Although height and weight were measured by trained health educators, other variables utilized self-reported survey components which may result in an under-reporting or over-reporting of pain symptoms. Our sample size was small. Since this was also a cross-sectional study, causation cannot be determined using these associations and statistical models. The reverse associations may occur in that musculoskeletal pain may affect obesity, as also supported by previous reviews [3,8,9,24]. Future studies should focus on testing the model in reverse and for causality, as well as obtaining a larger population for more pronounced relationships. Our measures of physical activity and physical function were limited, and had some overlaps in work and exercise allocations, which should be segregated in future questions. In addition, other factors have been proposed as possible mediators in the relationship between obesity and HRQOL outcomes, such as obesity-related comorbidities, and should be tested in addition to joint pain in future models.

## Conclusion

Our data indicate that being overweight and obese is an important correlate of impaired HRQOL, Physical Activity, and ESE. In addition, high-risk occupations such as the nursing home population have a high prevalence of musculoskeletal pain in WB and NWB joints. The results from the empirical test of the OMH and OEP model suggest that experiencing pain may be a mechanism by which obesity affects impaired HRQOL, lower PA levels, and lower ESE. Specifically, the WB joints seem to have a larger influence on vigorous physical activity and function, and NWB joints (particularly the wrist-forearm) have a larger influence on moderate physical activities and function. Lower ESE is also a significant partial mediator in the relationship between obesity and physical activity, particularly in moderate and vigorous physical activities, and should also be addressed in weight-loss programs for overweight and obese adults with or without joint pain.

## Figures and Tables

**Table 1:** General Demographic Characteristics of Participants (n=99).

Characteristic	N	n%
**Age**		
18–29	6	6%
30–49	51	52%
50–64	35	35%
>65	6	6%
**Gender**		
Male	9	9%
Female	90	91%
**BMI**		
Overweight	20	20.2%
Obese Class I	34	34.3%
Obese Class II	23	23.2%
Obese Class III	22	22.2%
**Education**		
Less than High School	8	8%
High School (Secondary)	39	39%
College/Professional	45	45%
Post-Graduate	7	7%
**Ethnicity**		
African American	40	40%
Caucasian	47	48%
Other	12	12%
**Job**		
Administration/Clerical	12	12%
CNA/GNA	30	30%
LPN	14	14%
RN	13	13%
Other	30	30%

**Table 2:** Associations between Level of Obesity, Health-Related Quality of life, Physical Activity, and Exercise Self-Efficacy.

	General Health	Physical Function (Moderate)	Physical Function (Vigorous)	Emotional Role (Accomplis h Less)	Emotional Role (Less Careful)	Social Interference	Current Physical Activity	Mild Physical Activity	Moderate Physical Activity	Vigorous Physical Activity	Exercise Self- Efficacy
**BMI** Correlation Coefficient	−0.191	−0.109	−0.308	0.137	0.004	0.005	−0.248	−0.129	−0.293	−0.176	−0.239
Significance (2-tailed)	0.059	0.284	0.002	0.178	0.969	0.961	0.013	0.202	0.003	0.082	0.017

* two-tailed p-value = 0.05,

** Bonferoni Correction p-value=0.005.

**Table 3:** Mediation Results on Moderate Physical Function in Participants (n=99).

	Path A	Path B	Path C	Direct Effect	Indirect Effect	Total Effect	Proportion Mediated	Bootstrapped 95% CI
				(Path C′)	(Bootstrapped Path A * B)	(Path C)	1-c′/c	(Lower Bound, Upper Bound)
**Mediator**	**Path Coefficient (b)**							
Low Back	0.1073	−0.1175	−0.042	−0.0294	−0.0117	−0.0411	0.3	−0.0473, 0.0122
Knee	0.0595	−0.0626	−0.042	−0.0383	−0.0031	−0.0414	0.0880952	−0.0274, 0.0135
Shoulder	−0.0482	−0.0865	−0.042	−0.0462	0.0041	−0.0421	−0.1	−0.0211, 0.0392
Wrist-forearm	0.2037	−0.1598	−0.042	−0.0094	−0.0341	−0.0435	0.7761905	−0.1050, −0.0036

**Table 4:** Mediation Results on Emotional Role (Accomplishing Less) in Participants (n=99).

	Path A	Path B	Path C	Direct Effect	Indirect Effect	Total Effect	Proportion Mediated	Bootstrapped 95% CI
				(Path C′)	(Bootstrapped Path A * B)	(Path C)	1-c′/c	(Lower Bound, Upper Bound)
**Mediator**	**Path Coefficient (b)**							
Low Back	0.1073	0.1966	0.1216	0.1005	0.0223	−0.0182	0.1735197	−0.0182, 0.0881
Knee	0.0595	0.0911	0.1216	0.1161	0.0072	0.1233	0.0452303	−0.0152, 0.0623
Shoulder	−0.0482	0.1023	0.1216	0.1265	−0.0032	0.1233	−0.0402961	−0.0592, 0.0218
Wrist-forearm	0.2037	0.2102	0.1216	0.0788	0.0434	0.1222	0.3519737	0.0010, 0.1520

**Table 5:** Mediation Results on Emotional Role (Less Careful) in Participants (n=99).

	Path A	Path B	Path C	Direct Effect	Indirect Effect	Total Effect	Proportion Mediated	Bootstrapped 95% CI
				(Path C′)	(Bootstrapped Path A * B)	(Path C)	1-c′/c	(Lower Bound, Upper Bound)
**Mediator**	**Path Coefficient (b)**							
Low Back	0.1073	0.1355	0.003	−0.0115	0.0155	0.004	4.8333333	−0.0117, 0.0711
Knee	0.0595	0.095	0.003	−0.0026	0.0075	0.0049	1.8666667	−0.0168, 0.0564
Shoulder	−0.0482	0.0736	0.003	0.0066	−0.0031	0.0035	−1.2	−0.0507, 0.0162
Wrist-forearm	0.2037	0.1712	0.003	−0.0318	0.0335	0.0017	11.6	0.0015, 0.0985

**Table 6:** Mediation Results on Exercise Self-Efficacy in Nursing Home Employees (n=99).

	Path A	Path B	Path C	Direct Effect	Indirect Effect	Total Effect	Proportion Mediated	Bootstrapped 95% CI
				(Path C′)	(Bootstrapped Path A * B)	(Path C)	1-c′/c	(Lower Bound, Upper Bound)
**Mediator**	**Path Coefficient (b)**							
Low Back	0.1073	−0.1212	−0.3377	−0.3247	−0.0152	−0.3399	0.0384957	−0.0949, 0.0109
Knee	0.0595	−0.0369	−0.3377	−0.3355	−0.0032	−0.3387	0.0065147	−0.0575, 0.0177
Shoulder	−0.0482	−0.0262	−0.3377	−0.339	0.0036	−0.3354	−0.0038496	−0.0206, 0.0478
Wrist-forearm	0.2037	−0.2925	−0.3377	−0.2781	−0.0583	−0.3364	0.176488	−0.1510, −0.0137

**Table 7:** Mediation Results on Physical Activity Levels in Nursing Home Employees (n=99).

	Path A	Path B	Path C	Direct Effect	Indirect Effect	Total Effect	Proportion Mediated by ESE	Bootstrapped 95% CI
				(Path C′)	(Bootstrapped Path A * B)	(Direct Effect + Indirect Effect)	1-c′/c	(Lower Bound, Upper Bound)
**Outcome Measures**	**Path Coefficient (b)**							
Current Physical Activity	−0.3635	0.116	−0.2567	−0.2145	−0.0428	−0.2573	0.1643942	−0.1348, −0.0001
Mild Physical Activity	−0.3635	0.0537	−0.1095	−0.0899	−0.0205	−0.1104	0.1789954	−0.0986, 0.0234
Moderate Physical Activity (PA)	−0.3635	0.1868	−0.2566	−0.1888	−0.0684	−0.2572	0.2642245	−0.1595, −0.0156
Vigorous Physical Activity (PA)	−0.3635	0.1743	−0.1415	−0.0781	−0.0631	−0.1412	0.4480565	−0.1365, −0.0171

## Abbreviations

ADL: Activities of Daily Living
BMI: Body Mass Index
ESE: Exercise Self-Efficacy
HRQOL: Health-Related Quality of Life
MS: Musculoskeletal
MCAR: Missing Completely at Random
MSD: Musculoskeletal Disorder
NWB: Non-weight-bearing
OA: Osteoarthritis
OEP: Obesity Exercise Self-Efficacy Physical Activity Model
OMH: Obesity Musculoskeletal Pain and Health-Related Quality of Life Model
ORCs: Obesity-Related Comorbidities
PA: Physical Activity
WB: Weight-bearing
WMSD: Work-related Musculoskeletal Disorders
